# 2’,3’-Cyclic Nucleotide 3’-Phosphodiesterases Inhibit Hepatitis B Virus Replication

**DOI:** 10.1371/journal.pone.0080769

**Published:** 2013-11-18

**Authors:** Hui Ma, Xing-Liang Zhao, Xue-Yan Wang, Xing-Wang Xie, Jin-Chao Han, Wen-Li Guan, Qin Wang, Lin Zhu, Xiao-Ben Pan, Lai Wei

**Affiliations:** Peking University People’s Hospital, Peking University Hepatology Institute, Beijing Key Laboratory of Hepatitis C and Immunotherapy for Liver Diseases, Beijing, P. R. China; Harvard Medical School, United States of America

## Abstract

2’,3’-cyclic nucleotide 3’-phosphodiesterase (CNP) is a member of the interferon-stimulated genes, which includes isoforms CNP1 and CNP2. CNP1 is locally expressed in the myelin sheath but CNP2 is additionally expressed at low levels outside the nervous system. CNPs regulate multiple cellular functions and suppress protein production by association with polyadenylation of mRNA. Polyadenylation of Hepatitis B virus (HBV) RNAs is crucial for HBV replication. Whether CNPs interact with polyadenylation signal of HBV RNAs and interfere HBV replication is unknown. In this study, we evaluated expressions of CNP isoforms in hepatoma cell lines and their effects on HBV replication. We found that CNP2 is moderately expressed and gently responded to interferon treatment in HepG2, but not in Huh7 cells. The CNP1 and CNP2 potently inhibited HBV production by blocking viral proteins synthesis and reducing viral RNAs, respectively. In chronic hepatitis B patients, CNP was expressed in most of HBV-infected hepatocytes of liver specimens. Knockdown of CNP expression moderately improved viral production in the HepG2.2.15 cells treated with IFN-α. In conclusion, CNP might be a mediator of interferon-induced response against HBV.

## Introduction

The 2’, 3’-cyclic nucleotide 3’-phosphodiesterase (CNP) belongs to the 2H phosphoesterase superfamily, which is characterized by the presence of two conserved HxT/Sx motifs (x denoting a hydrophobic residue) in the active site [1]. CNP contains an N-terminal domain that is distantly related to the P-loop containing nucleoside triphosphate hydrolases. The N-terminal domain is also involved in CNP dimerization and direct interaction with the calcium sensor calmodulin [2,3]. The C-terminus is a phosphodiesterase domain, which catalyzes the formation of 2’-nucleotide products from 2’,3’-cyclic substrates [4,5]. The isoprenylation of C-terminus mediates the binding of CNP to membranes and may regulate cytoplasmic microtubule distribution [6,7]. The activity of phosphodiesterase plays a key role in tRNA splicing in yeast. However, the substrate for CNP and its role in the life of mammals is unknown [8].

CNP is expressed as two isoforms, with CNP2 identical to CNP1 with a 20 amino acid extension at the N-terminus of CNP2 [9]. The 20-residue extension of CNP2 functions as a mitochondrial targeting signal that is controlled by phosphorylation [10]. CNP1 is locally expressed in the myelin sheath, and represents 4% of the CNS myelin proteins and 1% of the proteins in the peripheral nervous system myelin [11,12]. CNP1 is likely an important component of the cytoskeletal machinery that regulates the outgrowth in oligodendrocytes [7]. Additionally, CNP2 is also expressed at low levels outside the nervous system. The functional role and the significance of CNP2 in non-myelinating tissues are unknown. It has been demonstrated that the catalytic domain of CNP associates with polyadenylation of mRNA and suppresses translation *in vitro* [13]. Recently, CNP was reported as an interferon-stimulated gene (ISG) and induced by type I IFNs. A recent report showed that CNP contributed to interferon-induced inhibition of HIV production by binding to the HIV structural protein Gag and blocking HIV-1 particle assembly [12,14].

Hepatitis B virus (HBV), a hepatotropic virus, belongs to the family *Hepadnaviridae* which is responsible for infecting two billion people worldwide [15]. Upon infection, the viral genome is transported into the cell nucleus and converted into a covalently closed circular DNA. This serves as a template for the transcription of the four major viral RNA species, including preC/pregenomic, preS1, preS2/S and X mRNA. The viral mRNAs serve as the template for the translation of viral proteins in addition to the pregenomic RNA that is used for reverse transcription. The HBV genome is reversely transcribed by HBV polymerase within the viral nucleocapsids and is then enveloped by surface proteins to produce the Dane’s particles. All of the HBV RNAs are modified by the 3’ polyadenylation and this modification is crucial for HBV replication [16-18]. In this study, we investigate CNP expression in hepatoma cell lines and liver specimens, and determine whether the CNP modulates the HBV life cycle.

## Materials and Methods

### Plasmids and Constructs

The plasmid pUC18-HBV1.2 containing a 1.2-fold full-length wild type HBV DNA (Genotype C, Accession: AY040627) was previously constructed [19]. Plasmids expressing CNP (Accession: NM_033133) isoforms CNP1 and CNP2, named pCDNA5-CNP1 and pCDNA5-CNP2, were constructed using a strategy as described previously [20]. The forward primer containing a BamH I cleavage site and reverse primers containing a Not I cleavage site for CNP1 and CNP2 amplification are listed in Table 1. The PCR products were then digested with enzyme BamH I and Not I (Biolabs, Ipswich, Massachusetts) and inserted into the pCDNA5 vector (Addgene, Cambridge, MA) digested with the same enzymes. To construct the shRNA plasmids for CNP knockdown, four shRNA oligonucleotides were designed based on the public TRC shRNA library (www.broadinstitute.org/rnai/public/, clone ID: TRCN0000284938, TRCN0000273151, TRCN0000273152, TRCN0000273153). The Hpa I and Xho I sites were introduced at 5’ and 3’ end, respectively. The sense and antisense oligonucleotides were synthesized (SBS Genetech, Beijing, China) and annealed according to the protocol as described at the website above. The annealed oligonucleotides were inserted into a lentiviral vector pLL3.7 (Addgene) digested with the same enzymes [21]. The recombinant plasmids pLL3.7-shCNP were evaluated by knockdown assay and optimized sequence TRCN0000273151 is listed in Table 1. All of the recombinant plasmids were verified by sequencing and prepared using a QIAGEN EndoFree midi kit (Qiagen, Hilden, German).

**Table 1:** Sequences of PCR primers and CNP shRNA.

**No**	**Gene**	**Forward**	**Reverse**	**Site**
1	CNP1	5’-cgggatccatgtcatcctcagggg ccaaggac	5-’ttgcggccgcaatcatatgatggtggaggacggcaa	205-1410
2	CNP2	5’-cgggatccatgaacagaggcttct cccgaa	5-’ttgcggccgcaatcatatgatggtggaggacggcaa	145-1410
3	CNP2	5’-acagaggcttctcccgaaaaag-3’	5’-gaagacctggccggctttg-3’	149-772
4	tCNP	5’-g aagacctggc cggctttg-3’	5’-gaagacctggccggctttg-3’	318-772
5	Actin	5’-gcgcgaaatcgtgcgtgacatt-3'	5’-gatggagttgaaggtagtttcgtg-3’	699-930
6	CNP shRNA	5'-tgtgttctcaccaccacttatgctcgagcataag tggtggtgagaacactttttc-3'	5'-tcgagaaaaagtgttctcaccaccacttatgctcga gcataagtggtg gtgagaacaca-3'	1411(3UTR)

Forward primer sets 1 and 2, 3 and 4 share the same reverse primers. The underline indicates the cleavage sites of restriction enzymes.

### Cell culture and plasmids transfection

Human hepatoma cell lines HepG2, Huh7 and 293T were maintained in DMEM medium (Invitrogen, Carlsbad, CA) supplemented with 10% FBS (Invitrogen), 100 units/ml penicillin, 100 mg/ml streptomycin. The HepG2.2.15 cells, derived from HepG2 cells and integrated into chromosome with 1.3-fold HBV-DNA, were maintained in complete medium supplemented with 380 μg/ml G418 antibiotic (Sigma, St. Louis, MO). For DNA transfection, cells at 70-80% confluence were transfected with the plasmids by using Turbofect *in virto* reagent (Fermentas, Thermo scientific, Barrington, IL). Lentiviruses for knockdown of CNP were prepared using lentivirus packaging system according to the manufacturer’s protocol (ViraPower™ Lentiviral Expression Systems, Invitrogen).

### Liver specimens from CHB patients

Patients were recruited from outpatients at Peking University People’s Hospital that underwent liver biopsy as part of a standard medical evaluation. Patients met the following criteria: adults (18–65 years old), positive for HBsAg for more than 6 months, positive for HBeAg, HBV DNA >10^5^ copies/mL, elevated serum alanine aminotransferase value two to 10 times the upper limit of the normal range. We studied liver specimens from 3 patients prior to therapy. 

### Indirect immunofluorescence

Cells were fixed with phosphate buffered saline containing 2% paraformaldehyde and penetrated by incubation with phosphate buffered saline containing 0.1% Triton X-100. Cells were then blocked and incubated with rabbit polyclonal anti-CNP antibody (1:200, Proteintech, Chicago, IL), mouse monoclonal antibody against HBx or Tubulin (1:200, Abcam, Cambridge, UK). Bound primary antibody was visualized by Alexa Fluor 594-conjugated goat anti-rabbit IgG, Alexa Fluor 488-conjugated goat anti-rabbit IgG or Alexa Fluor 488-conjugated goat anti-mouse IgG (Invitrogen). Mitochondria were stained by cationic dye using a Mitocapture^Tm^ kit as manual (Merck Millipore, Billerica, MA). Cell nuclei were stained with 4',6-diamidino-2-phenylindole (DAPI, Invitrogen). Images were revealed by a fluorescent microscope (AX80TF, Olympus, Tokyo, Japan). For immunostaining of paraffin-embedded liver biopsies, deparaffinization and antigen retrieval was performed before the immunostaining as previous reported [22].

### Quantification for HBV DNA, HBsAg and HBeAg in culture medium

HBV DNA was quantified using a commercially available quantitative real-time fluorescence PCR kit (Piji Biotechnology Development Co., Shenzhen, China). The primers and probe for PCR are previously described [23]. For eliminating the residual plasmid pUC18-HBV1.2, the medium was digested with a final concentration of 200 μg/ml DNase I (Promega, Madison, WI, USA) in 10mM MgCl_2_ at 37 °C for 1 h, and the reaction was stopped with 25mM EDTA. Complete viral particles were isolated from cell supernatants by immunoprecipitation with a polyclonal anti-HBs antibody (Biodesign International, Kennebunk, Maine, USA) before HBV DNA quantitative analysis. Real-time PCR was performed on a LightCycler instrument (Roche, Mannheim, Germany). HBsAg and HBeAg in culture medium were detected using electrochemical illuminescent immunoassay kits (Abbott, Chicago, Illinois) on an ARCHITECT i2000 automatic immunoassay analyzers (Abbott).

### RNA isolation and qRT-PCR analysis

Total RNA (tRNA) was isolated using Trizol (Invitrogen) according to the manufacturer’s protocol. One μg of tRNA was then converted into cDNA by Transcriptor First Strand cDNA Synthesis Kit (Roche, Basel, Switzerland) with random hexamers as primers. cDNA was detected by quantitative real-time PCR using Faststar SYBR Green Master kit as manual (Roche). Primers for target mRNA were shown in Table 1. Twenty ng of tRNA was directly used as template to assess the possible DNA contamination in the RNA preparation. PCR conditions consisted of an initial denaturation step of 95°C for 5 minutes, followed by 40 cycles of 95°C for 20 seconds, 60°C for 30 seconds. The standard curve for CNP quantification was generated using serial 10-fold dilutions of linearized plasmid pCDNA5-CNP2. β-actin was used for reverse transcription and control for qRT-PCR.

### Viral DNA and RNA analysis

Intracellular viral DNA was extracted as described previously [24]. One-half of the DNA sample from each well of 12-well plates was resolved by electrophoresis into a 1.5% agarose gel and transferred onto a Hybond-XL membrane. For viral RNA analysis, total cellular RNA was extracted with TRIzol reagents (Invitrogen). Five micrograms of total RNA was resolved in a 1.5% agarose gel containing 2.2 M formaldehyde and transferred onto a Hybond-XL membrane in 20×SSC buffer (1×SSC is 0.15 M NaCl plus 0.015 M sodium citrate). For the detection of HBV DNA and RNA, membranes were probed with a whole genomic HBV-DNA probe labeled using a PCR DIG probe synthesis kit (Roche). The membrane was exposed to a X-Omat film (Kodak Company, Rochester, New York, USA), and hybridization signals were quantified with the QuantityOne software program (Bio-Rad).

### Western blot analysis

Cells were lysed with 1×Laemmli buffer. A fraction of cell lysate was separated on sodium dodecyl sulfate-12% polyacrylamide gels and transferred to a polyvinylidene difluoride membrane (Merck Millipore). Membranes were blocked with 0.05% TBST (Tris-Buffered Saline Tween-20) containing 5% nonfat dry milk and probed with antibodies against CNP (Proteintech 1:3000), β-actin (Abcam, 1:5000), secondary antibodies conjugated to horseradish peroxidase were used and followed by ECL (Merck Millipore) detection. The image was digitized using a scanner and signal was quantified using of QuantityOne software.

### Ethics Statement

The study was conducted in accordance with the ethical guidelines of the Declaration of Helsinki and was approved by the Ethics Committee of Peking University People’s Hospital. Written informed consent was obtained from each patient.

### Statistical Methods

Comparisons between two groups were carried out with the Student t-test and between multiple groups by one way analysis of variance. A *P* value of less than 0.05 was considered statistically significant unless otherwise noted.

## Results

### Characterization of CNPs in hepatoma cell lines

**Figure 1:**
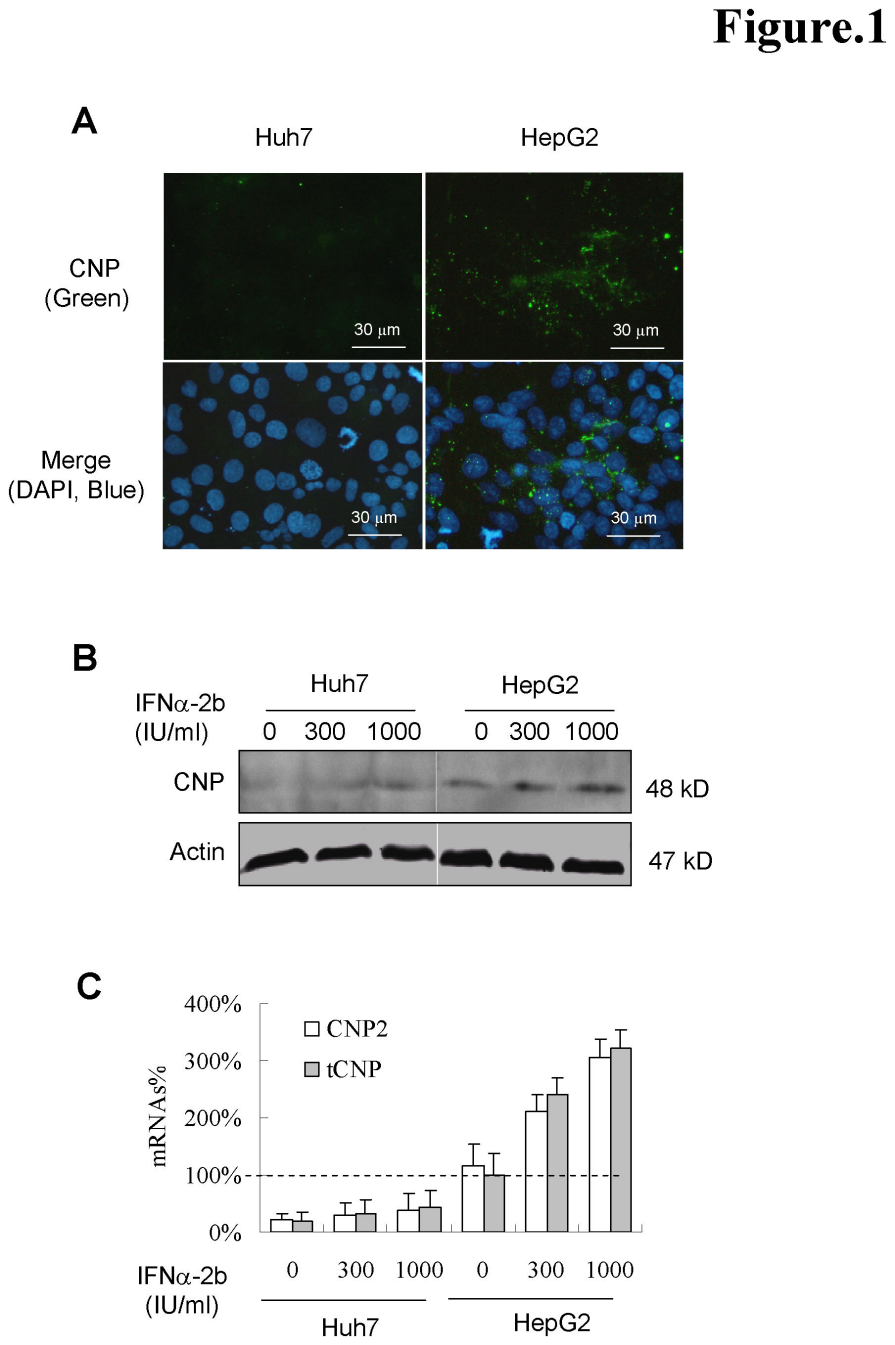
Native expression of CNP in human hepatoma cell lines. (A) The expression of the CNP proteins was studied by immunofluorescence microscopy using the anti-CNP polyclonal rabbit antibody and Alexa Fluor 488-anti-rabbit IgG. The nuclei were counterstained with 4,6-diamidino-2-phenylindole (DAPI). (B) Huh7 and HepG2 cells were cultured without or with 300, 1000 IU/ml of IFNα-2b for 2 days. Cells were harvested and the cell lysates were determined by Western blot analysis. Actin served as a loading control (protein panel). (C) For CNP mRNA quantitative analysis, cDNA pools were synthesized from the 1 μg of total RNA of cells and diluted cDNA was amplified by quantitative real-time PCR. Actin was served as reverse transcription and PCR control. The mRNA% presents the ratio of values of total CNP (tCNP) to that of naive HepG2 cells (n=3).

CNP2 is expressed at low levels outside the nervous system, suggesting a possible role in other organ [25]. To evaluate the CNP expression and its response to interferon in the hepatoma cell lines, we analyzed its mRNA and protein status in Huh7 and HepG2 cells. As shown in Figure 1A, immunocytochemistry showed moderate CNP expression in HepG2 but not in Huh7 cells. CNP expression was mostly found punctate and dispersed in cytoplasm of HepG2 cells. The CNP is detected at about 48kD, suggesting a CNP2 isoform (Figure 1B). To amplify the CNP2 form, which has a 60 base pair extension at the N-terminus, we designed the CNP2 forward primer spanning the N-terminus (shown in Table 1). Since CNP1 mRNA can not be differentiated from the CNP2 mRNA by PCR, we amplified the total CNP (tCNP) by reverse primers (Table 1) recognizing a shared region by both CNP1 and CNP2. Consistent with the observation by Western blot, CNP2 and tCNP were weakly detected in Huh7 cells. IFN-α poorly induced CNP expression in Huh7 cells. In HepG2 cells, a moderate CNP2 and tCNP expression were detected, with CNP2 comprising the tCNP levels. CNPs were moderately induced by interferon. One thousand IU/ml of IFN-α stimulated CNP2 and tCNP by about 3-fold in HepG2 cells (Figure 1C).

### Expression of CNPs potently inhibit HBV production

**Figure 2:**
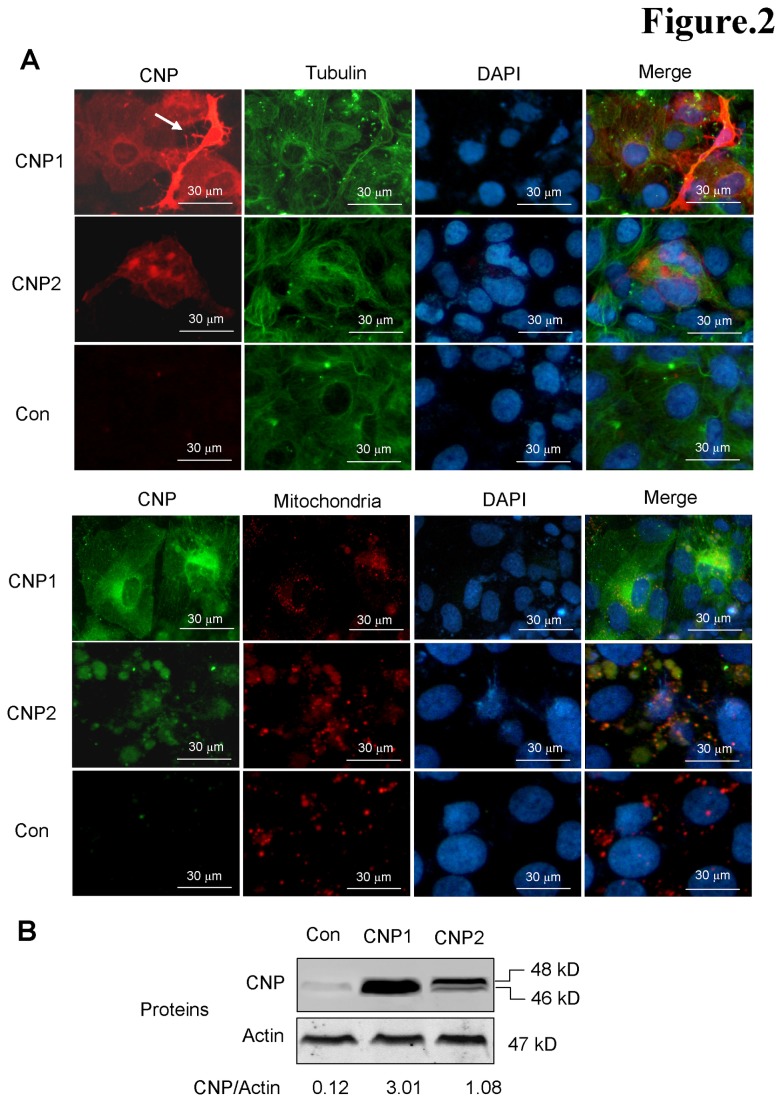
Subcellular location of CNP isoforms in Huh7 cells. (A) Huh7 cells were transfected with pCDNA5-CNP1 or pCDNA5-CNP2 and harvested at day 2 post transfection. The expression of CNP and tubulin was revealed by immunofluorescence microscopy using the anti-CNP polyclonal rabbit antibody and anti-tublin monoclonal mice antibody, the secondary antibodies were Alexa Fluor 594-conjugated goat anti-rabbit IgG Alexa Fluor 488-conjugated goat anti-mouse or rabbit IgG. The nuclei were counterstained with DAPI. Mitochondria were stained using Mitocapture^Tm^ kit and indicated by red color. White arrow indicates a CNP1-transfected Huh7 cell undergone a morphological change. (B) Huh7 ells in 12-well plate were transfected with 2 μg of plasmids pCDNA5-CNP1 or pCDNA5-CNP2. Cell lysates were determined by Western blot analysis. Actin served as a loading control.

Huh7 cells barely express CNP proteins, thus providing an ideal *in vitro* model to study the effect of exogenous CNP on HBV infection. Transfection of CNPs into Huh7 cells showed differential localization of CNP1 and CNP2 (Figure 2A). Consistent with previous observation [7,8], CNP1 exhibited a filamentous distribution in the cytoplasm, particularly congregated near the nuclei, and was co-localized with cytoskeleton microtubules. Interestingly, strong CNP1 expressing Huh7 cells undergone a morphological change characterized by an oligodendrocyte-like outgrowth (Figure 2A, upper panel, white arrow). CNP2 contains a mitochondrial-targeting signal at its N-terminus [4,10], and its expression co-localized with the mitochondria in the cytoplasm (Figure 2A, lower panel). By western blot, a 46 kDa band of CNP2 was also detected along with its canonical 48 kDa product (Figure 2B). This result confirms previous observations that CNP2 mRNA is able to produce both CNP1 and CNP2 polypeptides by alternate translation initiation codons [9]. 

**Figure 3:**
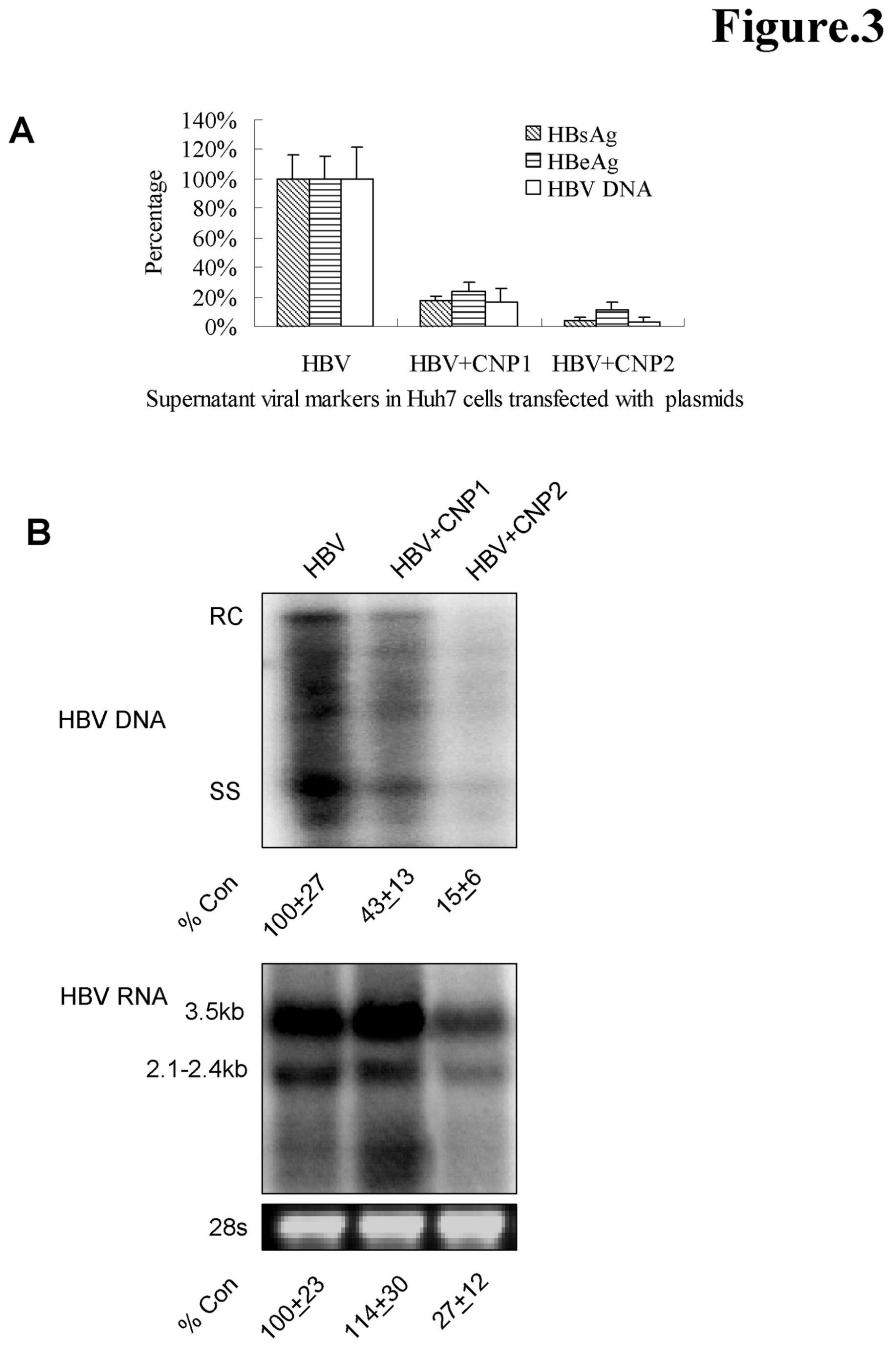
CNPs markedly inhibit HBV replication in Huh7 cells. (A) Huh7 cells in 12-well plate were co-transfected 1 μg of pUC18-HBV.12 with 1 μg of pCDNA5 vector, pCDNA5-CNP1 or pCDNA5-CNP2. 3 days post-transfection, supernatants were collected 3 days post-transfection for quantification of HBsAg, HBeAg using of electrochemical illuminescent immunoassay and HBV DNA was quantified using of qRT PCR (n=6). (B) Viral DNA and RNA were extracted and analyzed by southern blot and northern blot, respectively. HBV DNA (RC plus SS) and HBV prgenomic RNA (3.5kb) was quantified using the QuantityOne Software. %Con presents the ratio of values to that of Huh7 cells transfected with pUC18-HBV1.2 only (n=3).

To evaluate the potential inhibition of CNPs on HBV production, we co-transfected the pUC18-HBV1.2 plasmid with pCDNA5-CNP1 or CNP2 into Huh7 cells. In a series of six independent experiments, the expression of CNP resulted in a consistent decrease of secreted viral proteins and HBV DNA (Figure 3A). Moreover, CNP2 had a more potent effect than CNP1 on decreasing viral protein production and HBV DNA. To determine at which steps CNP inhibits viral life cycle, HBV replicative mediators were analyzed by northern and southern blot. Surprisingly, level of HBV RNAs slightly increased but HBV replicative mediators markedly decreased upon CNP1 expression, suggesting that CNP1 inhibits viral replication during post-viral RNA transcription. However, CNP2 was able to dramatically decreased levels of HBV RNAs, suggesting the markedly different functional mechanism between the different CNP isoforms (Figure 3B).

### Knockdown of Interferon-induced CNP increases HBV production

**Figure 4:**
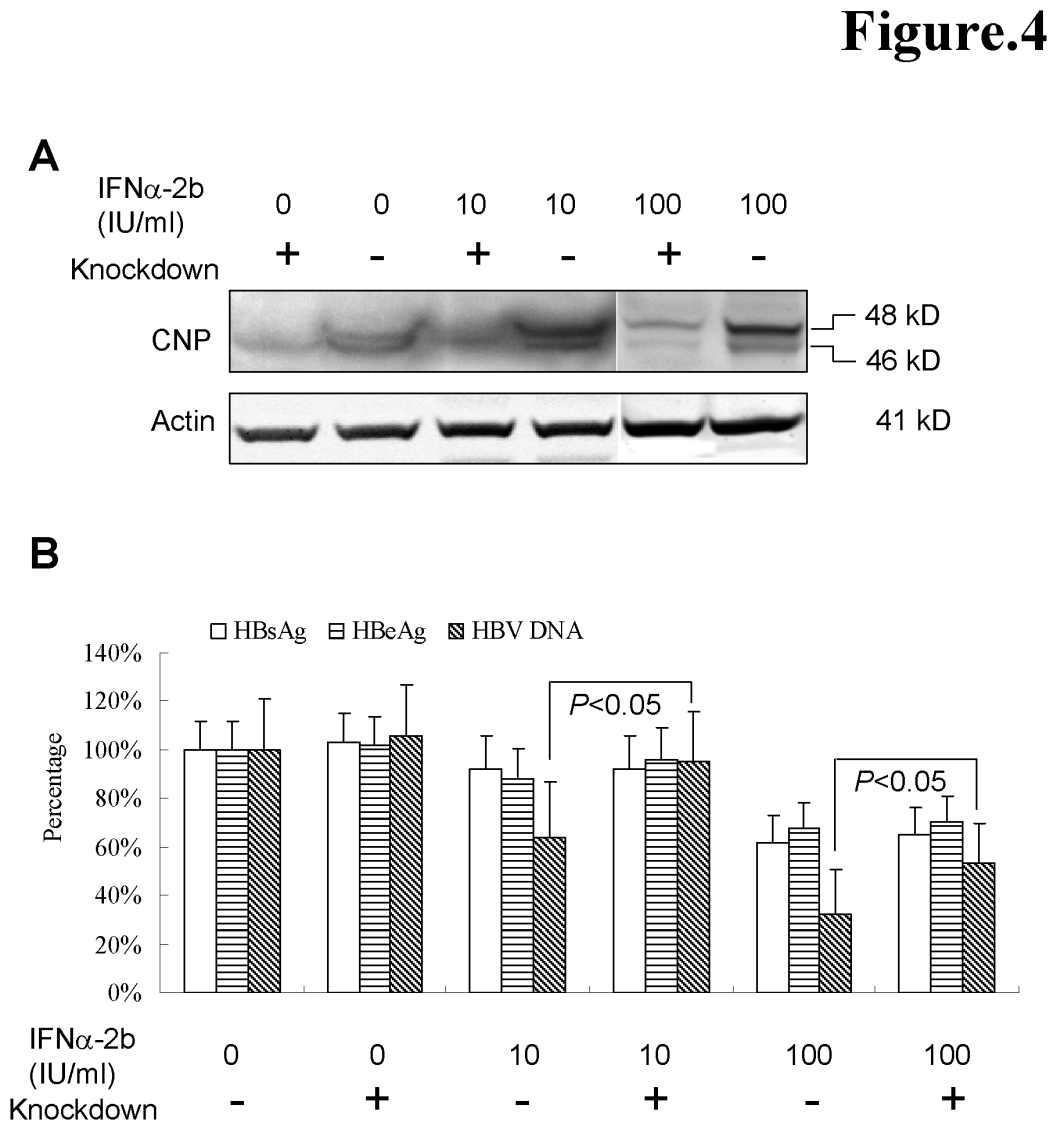
CNP is a functional mediator of IFNα-induced antiviral response against HBV. HepG2.2.15 cells were infected with lentivirus expressing shRNA of CNP at 10 MOI for 8 hours, and then medium was renewed by medium with indicated concentration of IFNα-2b for 3 days. (A). Cells were collected for analysis CNP expression by western blot. (B). Supernatants were collected for quantifications of HBsAg, HBeAg and HBV DNA. The value of percentage (Y axis of histogram) presents the ratio of HBsAg, HBeAg and HBV DNA obtained from other panels to control HepG2.2.15 panel (n=3).

 Over-expression of CNP potently inhibited HBV protein synthesis and virion production (Figure 3). It is unknown whether physiological expression or interferon induced CNP in hepatoma cell lines inhibits HBV replication. Hence, we knockdown CNP expression in the HepG2.2.15 cells with or without IFN-α treatment using pLL3.7-shCNP lentivirus (Figure 4A). In CNP knockdown HepG2.2.15 cells with interferon treatment, the cell-free HBV DNA was increased by about 20-30%. No changes were observed in control cells without interferon treatment (Figure 4B). These observations implied that although the native CNP expression might be not strong enough to inhibit HBV replication, interferon-induced CNPs might contribute to inhibition of HBV production.

### CNP is expressed in liver tissues with HBV infection

**Figure 5:**
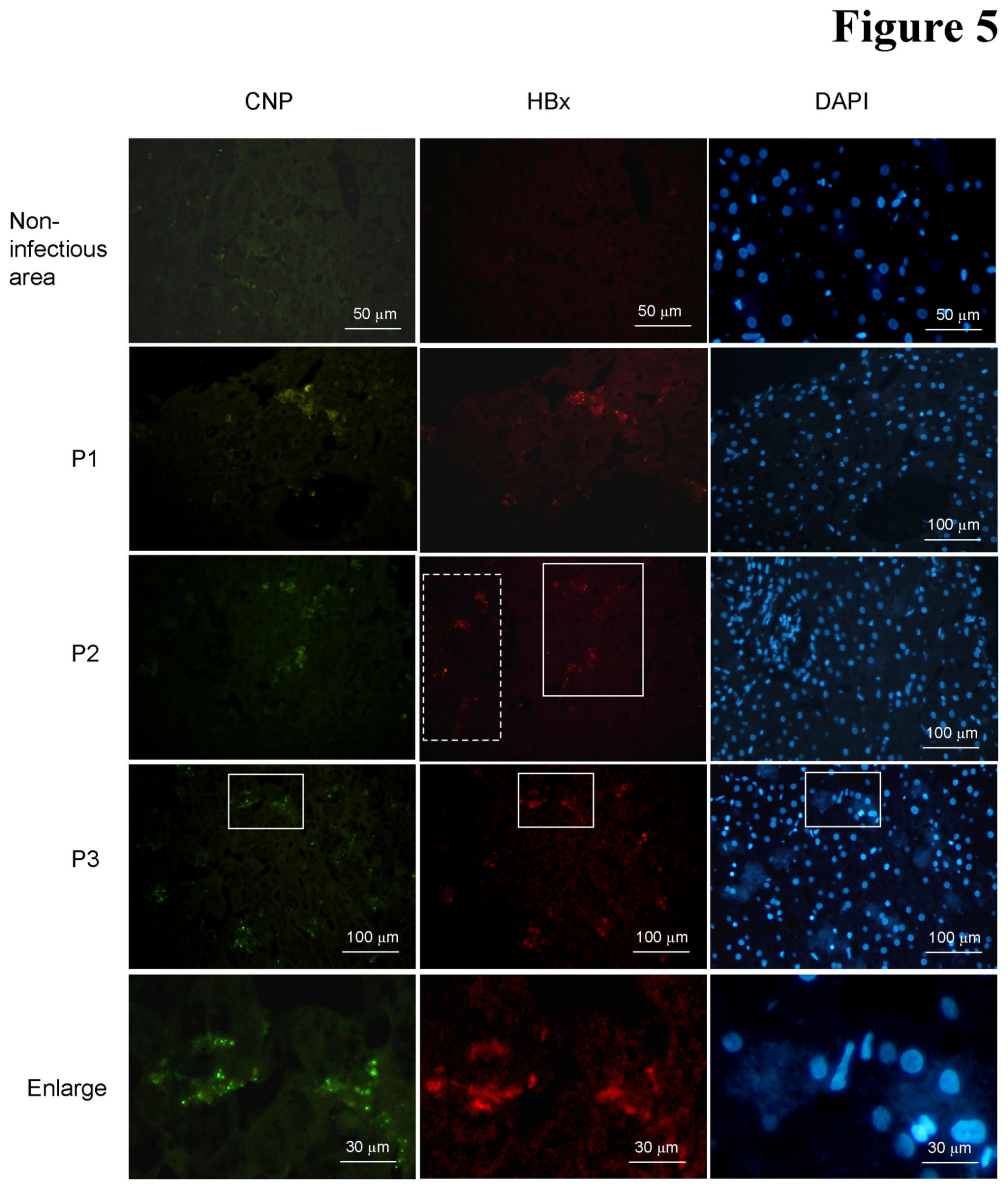
CNPs expression in the HBV-infected human liver specimens. Panels show micrographs of liver biopsy specimens from three HBV-infected patients. Specimens were costained with antibodies against HBV X protein (red) and CNP (green); nuclei were stained with DAPI (blue). These images were collected at 100 magnification and show the representative images. Control micrograph was collected from the non-infectious area. The dashed rectangle in P2 (Patient 2) indicated the HBx positive but CNP negative cells; the real line indicated the both positive area. The enlarge panel exhibits the subcellular distribution of HBx and CNP from the real line area of P3 panel.

To confirm whether CNPs express in hepatocytes *in vivo*, we analyzed the CNP expression in liver specimens from chronic HBV (CHB) patients. We used immunohistochemical staining and microscopy to assess the levels of CNP in sections of liver biopsies from CHB patients. HBV x protein was stained for indication of HBV-infected hepatocytes. Figure 5 shows the representative HBx and CNP expression in liver sections from 3 patients. CNP was weakly expressed in non-infectious regions of CHB, consistent with its reported low expression in the liver [26]. However, quantitative analyses of 100 HBV-infection foci from each section revealed that over 60% (62.4+15.8%) of HBx positive cells were expressed CNP protein, suggesting that CNP expression was markedly induced in HBV-infected hepatocytes *in vivo*. HBx mostly diffused in the cytoplasm and CNP was observed in a punctate manner. However, micrograph collected from cell sections could not confirm which CNP isoform was specifically expressed.

## Discussion

Although it has been shown that HBV can evade host innate immunity surveillance, the virus is sensitive to interferon-induced antiviral response in 30 to 40% of the treated patients [27-29]. Interferons restrict the replication of HBV by inducing the expression of interferon-stimulated genes (ISGs) that ultimately can result in the resolution of the chronic HBV infection [30-36]. In this study, we demonstrated that the ISG CNPs inhibit HBV production in hepatoma cell lines.

Firstly, we detected the expression of CNP isoforms in hepatoma cell lines. We found that CNP2 moderately expressed and gently responded to interferon treatment in HepG2, but not in Huh7 cells (Figure 1). This implies that the expression of CNPs might be heterogeneous in the hepatocytes of individuals. Furthermore, we demonstrate that the expression of both CNP isoforms potently inhibits HBV replication and viral protein synthesis. To our knowledge, this is the second virus that is able to be inhibited by CNP. However, the inhibition by CNP1 and CNP2 appear to have distinct mechanism. CNP2, but not CNP1, decreased intracellular HBV RNAs (Figure 2), suggesting that the isoforms targeted the different stages of the HBV life cycle. This result is consistent with previous observations that CNP1 associates with the poly(A)+ of mRNA and stabilize mRNA but suppresses translation initiation *in vitro* [13]. 

Although a 20 amino acid difference at the N-terminus between the CNP isoforms, CNP2 displays a distinct effect from CNP1. CNP2 markedly decreased intracellular viral RNAs by about 70% (Figure 3B). The 20-residue extension in the N-terminus of CNP2 serves as a mitochondrial targeting signal and translocates the CNP2 to the mitochondrial (Figure 2A). Whether the RNAs associated with CNP2 are degraded by universal RNase in the process of translocation in the cytosol or in mitochondrial is unknown. Further studies into the structure and function of CNP2 might provide insight as to how the 20aa at N-terminus might mediate this. Because CNP targets the poly(A) of mRNA, this protein exhibited a non-specific effect on protein synthesis. Transfection assays using series amounts of plasmids showed that CNPs, especially CNP2, also obviously inhibited expression of themselves (data not shown). The knockdown assay demonstrated that the interferon-induced CNP moderately inhibited HBV replication and viral protein production (Figure 4), suggesting that CNP might be a contributor of antiviral effect during the interferon therapy. Interestingly, although stronger inhibition of HBV replication was seen with higher interferon treatment, less CNP-mediated anti-HBV effect (about 20%) was observed upon CNP knockdown. This might be attributed by the antiviral effects of IFN is mediated by activation of several ISGs which might mask the effects of CNP. Furthermore, we observed that CNP expression was markedly elevated in the most HBV-infected hepatocytes in liver tissue (Figure 5). Whether this expression was caused by the inflammation-associated cytokines such as interferons or viral proteins is unknown. However, this phenomenon suggested that CNP expression is associated with HBV infection.

In summary, our results demonstrated that CNP isoforms, particularly the CNP2, might be a mediator of interferon-induced response against HBV. Furthermore, owing to CNPs regulates multiple cellular functions, it is worthy to note whether HBV replication-related CNP expression might contribute to disease progression of persistent HBV infection.
